# Methodological criteria for the assessment of moderators in systematic reviews of randomised controlled trials: a consensus study

**DOI:** 10.1186/1471-2288-11-14

**Published:** 2011-01-31

**Authors:** Tamar Pincus, Clare Miles, Robert Froud, Martin Underwood, Dawn Carnes, Stephanie JC Taylor

**Affiliations:** 1 Department of Psychology, Royal Holloway, University of London, London, UK; 2 Centre for Health Sciences, Institute of Health Science Education, Queen Mary University of London, UK; 3 Warwick Clinical Trials Unit, University of Warwick, Coventry, UK

## Abstract

**Background:**

Current methodological guidelines provide advice about the assessment of sub-group analysis within RCTs, but do not specify explicit criteria for assessment. Our objective was to provide researchers with a set of criteria that will facilitate the grading of evidence for moderators, in systematic reviews.

**Method:**

We developed a set of criteria from methodological manuscripts (n = 18) using snowballing technique, and electronic database searches. Criteria were reviewed by an international Delphi panel (n = 21), comprising authors who have published methodological papers in this area, and researchers who have been active in the study of sub-group analysis in RCTs. We used the Research ANd Development/University of California Los Angeles appropriateness method to assess consensus on the quantitative data. Free responses were coded for consensus and disagreement. In a subsequent round additional criteria were extracted from the Cochrane Reviewers' Handbook, and the process was repeated.

**Results:**

The recommendations are that meta-analysts report both confirmatory and exploratory findings for sub-groups analysis. Confirmatory findings must only come from studies in which a specific theory/evidence based *a-priori *statement is made. Exploratory findings may be used to inform future/subsequent trials. However, for inclusion in the meta-analysis of moderators, the following additional criteria should be applied to each study: Baseline factors should be measured prior to randomisation, measurement of baseline factors should be of adequate reliability and validity, and a specific test of the interaction between baseline factors and interventions must be presented.

**Conclusions:**

There is consensus from a group of 21 international experts that methodological criteria to assess moderators within systematic reviews of RCTs is both timely and necessary. The consensus from the experts resulted in five criteria divided into two groups when synthesising evidence: confirmatory findings to support hypotheses about moderators and exploratory findings to inform future research. These recommendations are discussed in reference to previous recommendations for evaluating and reporting moderator studies.

## Background

In many areas of health-related research, attention has started to focus on better matching sub-groups of patients to interventions. The aim of this is to improve the effectiveness of treatment and avoidoffering treatment to groups for whom treatment is neither acceptable, nor beneficial. In some areas, for example research in the musculoskeletal pain and mental health population, identification of sub-groups is considered a priority. Within the context of this consensus statement, the focus of sub-group analysis is on baseline factors that moderate treatment effects.

### Definitions

The definitions of moderators and mediators have been refined in recent years. We have adopted definitions from Kraemer and colleagues [1], which clearly describes how participant factors affect outcome:

a) Effect moderators represent variables, *e.g*. patient characteristics, measured at baseline that interact with treatment to change outcome for each sub-group. The interaction should be related to outcome in the linear model with or without a main effect. These specify for whom and under what conditions treatment is most effective, and can improve power in subsequent trials by better selection of target groups for stratification. The identification of sub-populations can also inform diagnostic theory and practice.

b) Predictors of treatment outcome are defined as baseline variables that affect outcome (significant main effect only) but do not interact with the allocated intervention. Such factors significantly predict outcome equally for target interventions and control conditions. A common example in multi-site trials is a significant difference in outcome between sites, without site-by-treatment effects.

c) Mediators are variables measured during treatment (such as change-in-process factors) that impact on outcome, with or without interaction with allocated treatment. Mediators help inform the process and potential mechanisms (including causal mechanisms through which treatment might work), and therefore can be used to improve subsequent interventions through strengthening the components that best influence the identified mediators. Mediators should not be a component of treatment or outcome. There should be a clear distinction between the constructs measured by the mediating variables and those of treatment and outcome.

### The aims of the consensus

Designing trials that include planned sub-group analysis depends, in the first instance, on informed decisions about such sub-groups (either from evidence or theory). Planned analysis necessitates complex design and large samples. It would be advantageous to systematically review published RCTs that have carried out sub-group analyses, or those that have provided sufficient information to carry out sub-group analysis by pooling data from several trials and identify the most promising findings to date. However, such reviews are compromised because of a lack of structured methodological quality criteria to guide the inclusion, synthesis and analysis. The Cochrane Handbook includes a section about sub-group analysis but does not provide list of criteria comparable to that provided for individual RCTs [2]. In addition, the interpretation of the advice given would result in a conservative approach to sub-group analysis, which is perhaps justifiable due to the risks association with this type of analysis [3]. Almost all current guidance on sub-group analysis at a meta-analytic level assumes that the decisions about carrying out such analysis will rest with the meta analysts; this ignores the possibility of pooling findings from trials that have independently carried out such sub-group analysis. The *a-priori *decisions about sub-grouping rest with the original trial researchers in these cases, rather than the meta-analysts. This consensus specifically addresses such cases.

The focus of this consensus study is on moderator analysis. The literature searches inevitably identified mediator analyses, but the criteria proposed below were developed to address moderators only.

As a starting point we assume that a typical systematic review of trials will include the assessment of all appropriate trials for specific target populations undergoing particular interventions. This assumption follows the Cochrane recommendations for literature search, inclusion criteria, assessments of risk and bias, data extraction, data analysis, and grading of evidence [2,4]. While these guidelines have been widely implemented for assessing trials, considerably less is known about the acceptability and utility of sub-group analysis within RCTs, and there is little guidance for researchers who wish to pool data from trials that have already carried out sub-group analysis. The aim of this consensus was to provide guidance about inclusion and exclusion of studies into such a review. We recognise that there is a need for guidance on statistical pooling of interactions from sub group analysis in RCTs, but this was beyond the scope of the current study. Our objective was to provide researchers with a set of additional criteria that will allow grading of evidence for moderators. Broadly, we aimed to achieve a consensus on methodological criteria for the evaluation of studies that have carried out moderator analysis in the context of RCTs.

### Associated literature

There are several excellent publications that discuss issues associated with the methodology and analysis of sub-groups (including moderators of treatment effects) in the context of trials. Since the publication of Baron and Kenny's seminal paper in 1986 [4] discussing moderation and mediation effects, there has been progress towards consensus on definition and process of good methodology. Some publications focus on providing advice for those planning moderator (or mediator) analysis in the context of new trials (*e.g. *[5]); some address the shortcomings and pitfalls inherent in current analytical approaches [6-11], and several focus on the problems inherent in calculating power and adequate sample size for moderator analysis [12-14]. Other publications focus on correct analytical methodology to differentiate or combine mediation and moderation effects (*e.g. *[15]), and a further group of publications relates the methodological issues associated with moderator/mediator analysis to specific groups of patients,( *e.g. *[16,17]). There is also sufficient guidance on carrying out systematic reviews in specific populations (*e.g *[18]). What is still missing is a set of agreed methodological criteria for assessment of studies that have carried out moderator analysis within RCTs, so that meta-analysts can make decisions about the eligibility of studies, and the interpretation of their findings within a context that is agreed and understood by other researchers.

## Method

### Development of the criteria

We identified theoretical papers focusing on moderator analysis by using a snowballing technique, and electronic database searches:

Searches of PubMed and Web of Science could not be used effectively, as the focus of the search was to identify theoretical publications on moderator analysis, and neither PubMed or Web of Science index allow such a search. The closest Medical Subject Heading (the NLM vocabulary) term is 'Effect Modifiers', but this only yielded publications in which moderator analysis were carried out rather than methods papers. We therefore did a further search in Google Scholar using a combination of keywords (sub-group analysis, trials, effect modification, moderator analysis). Finally the reference sections of papers known to the authors were searched to identify other relevant papers. Reference sections of these were subsequently searched

**Figure 1:**
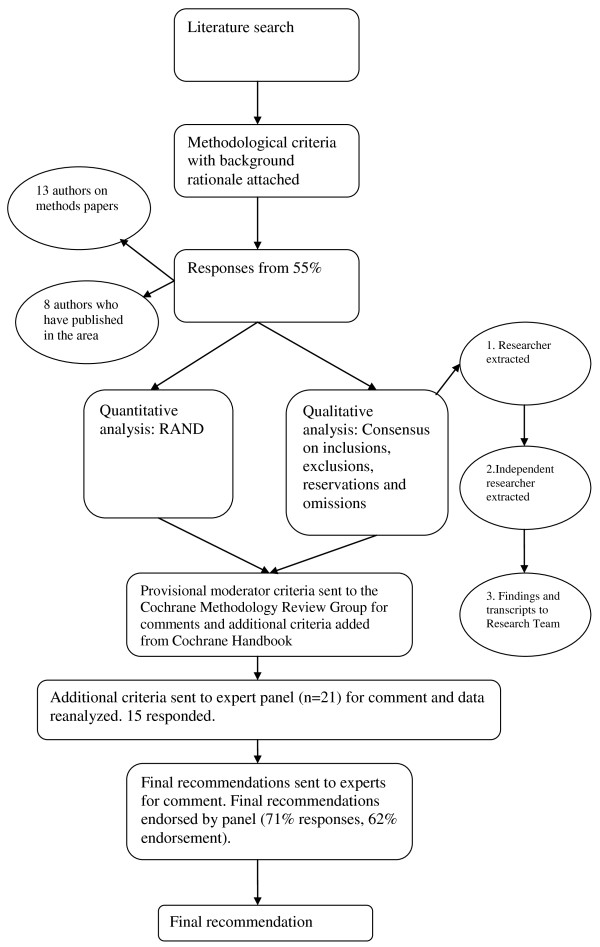
**The study process**.

We produced a synthesis of criteria indicated either by theoretical publications, publications based on simulated data, and publications on studies demonstrating theoretical points through secondary analysis of data from RCTs. From these publications, we extracted 11 potential criteria to be reviewed by the consensus panel. We presented a rationale for each criterion, wherever possible using verbatim quotes from published research (see Table 1). The coding sheet presented each criteria against a rating scale (ranging from 0 = completely inappropriate, to 8 = extremely important). It was necessary to use a 9-point scale in order to facilitate our analysis of appropriateness and disagreement using established Research ANd Development (RAND/UCLA) methodology (*see appendix*) [19-21]. The list of criteria and the rationale were then e-mailed to a panel of selected experts (Tables 1 and 2). After synthesising the responses from the consensus panel, we compared our list of criteria directly to the recommendations from the Cochrane Handbook [2]. We extracted four additional criteria that were missing from our original list, and repeated the consensus process on these criteria. The complete process is described in figure 1.

**Table 1:** Methodological criteria and rationale

Criteria	Rationale
Stage 1:	

1) Rationale:	
a) Was the analysis *a- priori *(planned in protocol rather than *post- hoc*)?	The need for a theoretical basis for choice of measurement to be tested as moderator or mediator. Ideally, the planned analysis is *a- priori*.
b) Was selection of factors for analysis theory/evidence driven?	**"**Ideally, these hypotheses are initially theory driven, then empirically confirmed, and finally clinically evaluated to establish their real-world existence." Nicholson et al., 2005

2) Method:	
a) Was there an equal distribution of moderators between groups at baseline?	Ideally, a-priori stratification in design (Lipchick et al., 2005, Headache).
b) Were moderators measured prior to randomisation?	"...a hypothesized moderator must be measured prior to randomization " Nicholson et al., 2005 page 516)

3) Power:	
Do authors report a power analysis for moderator effect (*a-priori *or *post-hoc*, but using an a-priori ES, not the observed one?	**"**In planning a test for the effect of a moderator, the researcher must pre-specify the size of a moderator effect that is deemed to be substantively important. The power calculation determines whether the statistical test proposed to detect the moderator effect has sufficient power... Retrospectively, power analyses may be used to evaluate moderator analyses that have already been conducted, by providing information about how sensitive the statistical tests were to detect substantively meaningful effects. If tests for moderator effects are found to have low power, statistically non-significant effects of moderators do not provide strong evidence for ruling out moderator effects. Alternatively, if a test for a moderator is found to have very high statistical power to detect the smallest substantively meaningful effect, then failure to detect that effect is evidence that moderator effects are not likely to be large enough to be substantively meaningful.
	In the retrospective application of power analysis, as in the prospective one, the researcher must pre-specify the size of a moderator effect that is deemed to be substantively important. That is, the moderator effect size must be determined a- priori. In particular, the observed effect of the moderator variable should never be used to compute statistical power" (extracted directly from Hedges & Pigott, 2004, page 427)
	Sufficient power to detect small/moderate effects in moderator analysis has been defined as at least four times that of the main effect (based on the fact that most main effects are in this order of magnitude).
Was sample size adequate for the moderator analysis (at least 4 fold the required sample size for main treatment effect in the lowest sub-group for the moderator factor)?	**"**The ability of formal interaction tests to (correctly) identify sub-group effects improved as the size of the interaction increased relative to the overall treatment effect. When the size of the interaction was twice the overall effect or greater, the interaction tests had at least the same power as the overall treatment effect. However, power was considerably reduced for smaller interactions, which are much more likely in practice. The inflation factor required to increase the sample size to enable detection of the interaction with the same power as the overall effect varied with the size of the interaction. For an interaction of the same magnitude as the overall effect, the inflation factor was 4" (HTA 2001 5 (33).
If not, were there at least 20 people in the smallest sub-group of the moderator?	An inherent problem is that power in RCTs is almost always calculated based on the main effect of treatment. Arbitrary cut-point has been used by other systematic reviews of at least 10 in lowest arm of completed treatment (Eccelston et al., updated for Cochrane, 2009.) We have included this arbitrary criterion to ensure retention of studies that were under-powered in isolation but might still add value to meta-analyses. However, with sub-groups below 20, we considered the study to be unlikely to be informative.
Have authors employed analysis to compensate for insufficient power (i.e. boot-strapping techniques?)	This criterion was included because some researchers attempt such analysis, and its value is debatable.

4) Correction for multiple comparisons:	
a) Was the regression significant at P < 0.05, or (if more than three comparisons) corrected or significance adjusted to P < 0.01?	In the absence of a-priori stratification, studies often explore several sub-groups, and the risk of type I error is considerably increased. The adjustment of P values has been used in RCT analysis (Turner, 2007).
b) Did the authors explore residual variances of interactions if carrying out multiple two-way interactions?	Multiple two-way interactions In many studies, researchers evaluate two or more moderators in a single analysis. For example, a regression model might include terms for XZ, WX, and WZ. Researchers sometimes make inferences about relative importance when, say, one of the three interaction terms is statistically significant and the others are not. However, such inferences require equivalent statistical power for each test. It might well be the case that the interaction terms are equivalent in terms of the sizes of their partial regression coefficients but that there are differences in statistical reliability due entirely to differences in the residual variances of the interactions terms. Thus when examining multiple two-way interactions, one ought to compare the residual variances of those interactions before making any inferences about their relative importances (McClelland & Judd, 1993, page 385)

5) Measurement validity & measurement error: Was measurement of baseline and process factors reliable and valid (from published information) in target population?	Measurement error considerably inhibits the power of studies to detect interactions.
a) Is there evidence that the measurement error of the instrument is likely to be sufficiently small to detect the differences between sub-groups that are likely to be important?	**"**Estimates of the reliability of measures should be reported. Measurement unreliability in M would be expected to lead to underestimating *b*' and thus *ab*', and overestimating *c*' (R. Baron & Kenny, 1986; Hoyle & Kenny, 1999)." Gelfand et al., 2009 p168
b) Did the authors comment on measurement validity in reference to construct validity, face validity etc?	"Trafimow (2006) described a concern for the construct validity of measures that is roughly analogous to that raised by measurement unreliability but for which there is currently no means of correction." Gelfand et al., 2009 p169.

6)Analysis:	
a) Contains an explicit test of the interaction between moderator and treatment (e.g. regression)?	**"**Sub-group analyses should always be based on formal tests of interaction although even these should be interpreted with caution." HTA, 2001
b) Was there adjustment for other baseline factors?	
c) Is there an explicit presentation of the differences in outcome between baseline sub-groups (e.g. standardised mean difference between groups, Cohen's *d*).	

Stage 2:	

1. Differences between sun-groups should be clinically plausible.	Selection of characteristics should be motivated by biological and clinical hypotheses, ideally supported by evidence from sources other than the included studies. Subgroup analyses using characteristics that are implausible or clinically irrelevant are not likely to be useful and should be avoided. " Section 9.6.5.4
2. Reporting of sub-group analysis is only justified in cases where the magnitude of the different is large enough to support different recommendations for different sub-groups.	"If the magnitude of a difference between subgroups will not result in different recommendations for different subgroups, then it may be better to present only the overall analysis results." Section 9.6.6
3. Within study comparisons are more reliable than between study comparisons.	"For patient and intervention characteristics, differences in subgroups that are observed within studies are more reliable than analyses of subsets of studies. If such within-study relationships are replicated across studies then this adds confidence to the findings. " Section 9.6.6
4. At least ten observations should be available for each characteristic explored in sub-group analysis (i.e., ten studies in a meta analysis).	"It is very unlikely that an investigation of heterogeneity will produce useful findings unless there is a substantial number of studies. It is worth noting the typical advice for undertaking simple regression analyses: that at least ten observations (i.e. ten studies in a meta-analysis) should be available for each characteristic modelled. However, even this will be too few when the covariates are unevenly distributed. Section 9.6.5.1

### Selection of the panel

We selected the panel of experts based on two considerations: The first group contained authors who had published methodological papers specifically on conducting sub-group analysis in the context of an RCT (n = 21 contacted, 13 responded). To avoid the potential bias of consulting only with those whose work was the basis for the criteria list, we also included a group of researchers who have been active in the study of sub-group analysis in RCTs. These researchers were identified from publications and conference presentations (n = 15 contacted, 8 responded). The final panel (n = 21) included epidemiologists (n = 5), biostatisticians (n = 3), clinical and public health researchers (n = 6) and researchers in social science (n = 7), and the contributors' nationalities comprised Australia (n = 3), Canada (n = 1), Netherlands (n = 3), UK (n = 5) and USA (n = 10). We requested ratings for each criterion, free comments on reservations, omissions, and redundancies.

### Coding

#### Quantitative analysis

In order to assess appropriateness of each item for recommendation and if there was disagreement between panel members, we used Research ANd Development (RAND)/University of California Los Angeles (UCLA) appropriateness method [18-20].The same method has since been used to measure consensus in a Delphi study [22]. Details of the method are provided in Appendix I.

#### Analysis of free responses

The free responses were tabulated so that comments on each criterion were categorised under the headings: reservations, omissions, and redundancies. These were also independently coded for accuracy of the categorisation, consensus and disagreement in reference to each criterion. The synthesis of this coding was then presented, together with the original table of quotations, to three members of the research team. All disagreements were discussed until consensus was achieved.

This study did not require ethical approval.

## Results

The electronic literature search on 'effect modifiers' resulted in 2,882 hits, but the majority of these were non-theoretical studies. The snowball approach resulted in 18 methodological publications, from which we extracted the items presented to the panel in the first stage.

### RAND analysis

Stage 1: Seventeen participants (47% of those contacted, 85% of responders) rated the presented items and provided usable quantitative data for analysis. There was no disagreement on the following: 1a) The analysis should be *a-priori *(planned in protocol rather than *post-hoc*); 1b) The selection of factors for analysis should be theory/evidence driven; 2b) Moderators should be measured prior to randomisation; 5a) Measurement of baseline factors should be reliable and valid; 6a) Analysis should contain an explicit test of the interaction between moderator and treatment (*e.g. *regression). Items 1a, 1b, 2b, 5a, & 6a are considered 'important' for inclusion in systematic reviews of moderator analysis in RCTs (Table 2). There was 'uncertainty' on the remaining items. No items were felt to be inappropriate.

**Table 2:** Median, 30^th ^centile, 70^th ^centile, appropriateness, and disagreement index, by item

	Median	**30**^**th **^**centile**	**70**^**th **^**centile**	Appropriateness	Disagreement index *
Stage 1					

1a	6.0	6	7	Appropriate	0.16

1b	6.5	6	8	Appropriate	0.29

2a	4.0	3	5	Uncertain	0.85

2b	8.0	7	8	Appropriate	0.13

3a	5.5	5	6	Appropriate	0.22

3b	4.5	3	6	Uncertain	0.97

4a	4.5	3	6	Uncertain	0.97

4b	5.0	4	5	Uncertain	0.32

5a	6.5	6	8	Appropriate	0.29

5b	5.0	3	6	Uncertain	0.97

6a	8.0	8	8	Appropriate	0.00

6b	4.5	3	6	Uncertain	0.97

6c	6.5	5	7	Appropriate	0.37

Stage 2					

1	6.0	4	7	Appropriate	0.65

2	0.0	0	5	Inappropriate	1.09

3	6.0	5	6	Appropriate	0.29

4	4.0	2	2	Uncertain	0.96

*A disagreement index of less than 1.0 indicates no disagreement

**Figure 2:**
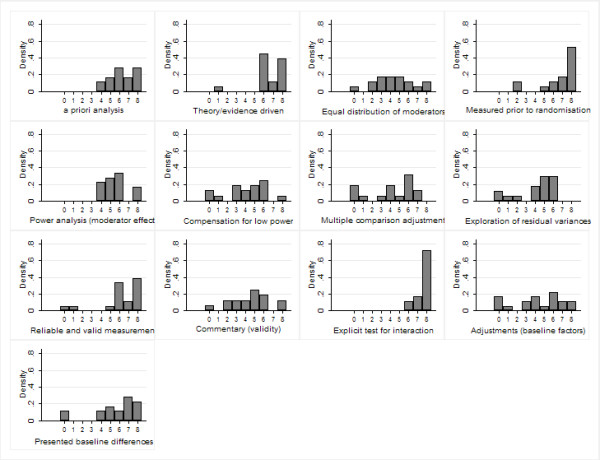
**The distribution of participants' ratings for each of the items (stage 1)**.

Figure 2 shows the distributions of participants' ratings for each of the items. Denser distributions toward the right of the scale indicate greater appropriateness. Leptokurtic (tall and thin) distributions, such as that shown for item 6a, indicate greater agreement; whereas platykurtic (flatter) distributions, such as that shown for 3b, indicate less agreement.

**Figure 3:**
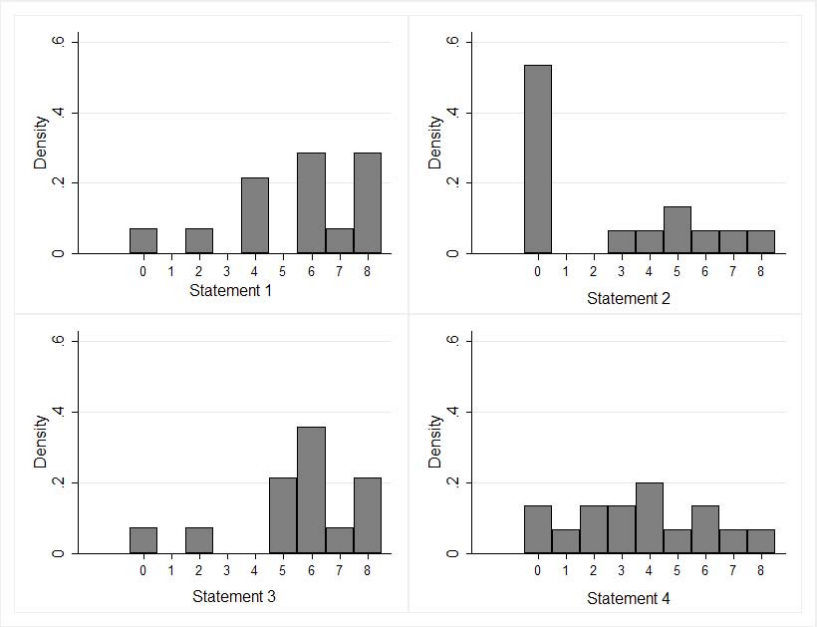
**The distribution of participants' ratings for the additional items (stage 2)**.

Stage 2: Fifteen participants (71% of those contacted for stage 2, based on all those who responded at stage 1) rated four additional items. There was agreement that two items were appropriate as recommendations: The panel agreed that sub-group analysis should be clinically plausible, and that evidence from high quality within-study comparisons is more reliable than between-study comparisons. The panel was uncertain about the recommendation that at least ten observations should be available for each characteristic explored in sub-group analysis *(i.e. *10 studies in a meta-analysis). The panel disagreed about the recommendation that sub-group analysis is only justifiable in cases where the magnitude of the difference is large enough to support different recommendations for different sub-groups. Figure 3 shows the distributions of participant's ratings for each of the items in stage 2.

#### Free responses

We received comments from 14 respondents.

#### Comments on criteria endorsed as necessary for inclusion in systematic reviews of moderators in RCTs

### Reservations

Six panelists expressed reservations about applying the criterion for inclusion of *a- priori *hypothesis testing as a necessary criterion for inclusion in a review. All six expressed a strong preference for *a- priori *planning at the protocol stage for moderator analysis, but felt that *post-hoc *testing should also be included in reviews, albeit with caution. The reasons given included that most data from trials are currently under-tested, and that post-hoc analysis can help identify factors for future evaluation through prospective studies. A strong recommendation emerged to report *a-priori *and *post-hoc *analyses separately, and describe the former as confirmatory testing and the latter as exploratory (*i.e*., hypothesis generating). Two panelists expressed reservations about inclusion of only theory/evidence driven moderators because the rationale for the decision is inherently subjective and in the absence of evidence or theory such analysis could still be informative, whilst being exploratory. Two panelists commented on the criterion stating measurement of moderators must be carried out before randomisation: They agreed that this was essential except in cases in which the moderator factor does not change over time (*e.g*. sex), and one panelist proposed that post-randomisation measurement may be acceptable if blinded. Five panelists agreed that well defined, reliable and valid measures, both for moderators and outcomes were essential or necessary for inclusion in systematic reviews of moderator analyses. There was some inconsistency about how to establish whether moderators and outcomes are well-measured, with recommendation that meta-analyses provide clear protocol. There was also a recommendation from one panelist that meta-analysts establish clear protocol to ascertain that moderators, treatment and outcome relate to the same constructs in all studies. Similarly, there was a recommendation that precise definitions of the target population applied across included studies should be specified. There was a strong endorsement by six panelists of the criterion stating that studies must include an explicit test of the interaction between moderators and treatment. Despite the endorsement of the criterion stating sub-group analyses should be clinically plausible, four panelists expressed reservations, since what may be regarded as clinically plausible depends on the current state of knowledge. Finally, several panelists supplied explanations for their endorsement of the statement suggesting that within-study comparisons are more reliable than between-study comparisons. This was because between-study comparisons suffer from additional sources of variation and the advantage of randomisation is lost. In addition, the study population in within-study comparisons is likely to be more homogeneous. However, three panelists also expressed the opinion that this depends, largely on the quality of single studies, and whether there is robust evidence of an effect across a study population.

#### Comments about criteria about which the panel felt uncertain

Six panelists agreed unequal distribution of moderators between the two randomised groups at baseline should not be considered a methodological weakness. They pointed out that although, theoretically, randomisation eliminates unequal distributions when N is large, in practice this may not occur for every baseline factor measured when N is limited. A cautionary note to authors, was that unequal distributions reduce power.

Five panelists commented that the criterion assessing reporting of a post-hoc power analysis may be redundant. While they agreed that in ideal circumstances trials will include an *a- priori *moderator hypothesis accompanied with a sample size analysis, they felt that issues surrounding calculation for *post-hoc *moderator analyses are considerably more complex. One panelist recommended that the choice of adequate power for inclusion should be set *a-priori* by the meta-analyst, using a threshold of clinical significance based on effect size.

Three panelists rejected the idea of including only studies that had at least four-fold the required sample size for the main effect, or a minimum of 20 participants at follow-up in each arm. They described criteria based on absolute sample size as unsupportable due to differing design requirements across trials. In such circumstances they suggest that inflating the number of participants by four would be both impractical and unethical.

Five panelists were in agreement that no consideration should be given to methods employed to compensate for insufficient power. Five panelists expressed opinions about multiple testing of moderators, and adjusting analyses to compensate for multiple testing. There was consensus that inclusion of multiple tests should rest with the reviewer based on *a-priori *hypotheses. Thus, multiple moderators may be tested in combinations to explore complex clinical prediction rules and against complex combinations of outcome measures, as long as the tested prediction are specific. However, reporting on a variety of tests with no clear rationale, or including a lengthy *a-priori* list of hypotheses for testing, may result in the study being included in the 'exploratory' group, rather than the 'confirmatory' group. There was also consensus that exact P-values were not informative in determining the clinical and practical significance of the finding, nor was there a need for presentation of standardised mean differences in outcome between the baseline sub-groups. Consensus was that the correct reported statistics should include a pooled effect size for the interaction with 95% confidence intervals. Four panelists expressed the opinion that adjustment of the P-value was erroneous and four panelists commented that in the context of RCTs (unlike observational studies) adjustment for other baseline factors may compromise randomisation and reduce power. There was disagreement about the value of exploring residual variances of interactions in multiple testing: while one panelist thought this would be informative for understanding power issues, two thought it was uncommon practice in RCT analysis and should not be included

The panelists were uncertain about the need to have at least 10 observations (separate studies) to test each moderator in a meta-analysis. Two panelists agreed in principle, but four regarded this as too simplistic/conservative, and supported carrying out meta-analyses with fewer studies (between 2 to 8).

The quantitative analysis showed that the panel disagreed about the criteria suggesting that reporting of sub-group analysis is only justified in cases where the magnitude of the difference is large enough to support different recommendations for different sub-groups. However, free responses suggested that the majority of the members did not support this criterion. Ten panelists expressed the opinion that once sub-group analysis was carried out, it must always be reported. Panel members commented that any analysis specified in a protocol must be reported regardless of findings, and that failure to do so would distort the literature through selective reporting. One panelist agreed with the statement and provided no free text.

### Comments on omissions

One panelist suggested that the checklist should include an assessment of sufficient variability of the moderator.

## Discussion

We have produced a consensus of methodological criteria for assessment of moderators in the context of systematic reviews of RCTs that carried out sub group analysis. This consensus is novel in that it provides clear evaluative criteria for systematic reviews that examine RCTs and have reported sub-group analyses. Current guidelines, such as those outlined in the Cochrane Reviewer's Handbook, focus on pooling data from studies in order to carry out sub-group analysis, rather than pooling data from studies that have already carried out such analysis. The recommendations from this consensus are that meta-analysts report both confirmatory findings for sub-groups, and exploratory findings, which can be used to inform further trials. We regard these recommendations as a first step and hope that they generate further discussion. Like all guidelines, these should be amended to reflect new knowledge and advances. We emphasise that those implementing criteria to assess moderators should always follow the normal quality criteria applied to RCTs to ensure sufficient methodological rigour in included trials [2,4]. In addition, we advise careful consideration for a) adequate similarity of intervention content, delivery and structure between studies; b) outcome measures that aim to assess the same underlying concept; and c) baseline variables measuring the same underlying construct. For b) & c), if individual participant data are obtained from different studies, one can consider analysing standardised scores if different measurement has been used, providing there is evidence that different instruments measure the same latent construct. We also wish to emphasise (reinforcing a strong sentiment from the consensus panel) that the study of moderators in RCTs is relatively new, and applying standards that are too rigid, could impede progress; especially for exploratory analysis. In addition, because almost all published trials fail to carry out a specific power analysis and sample size calculation for testing moderators, lack of power is ubiquitous, and absence of statistical significance should not be regarded as evidence of absence of an effect [23]. These criteria will help guide researchers on how to approach the selection of trials, that have carried out subgroup analysis for inclusion in a meta-analysis. Our recommendations build on those made previously [2,24,25] in that they suggest criteria for confirmatory findings. Previous recommendations did not specify the circumstances in which results could be regarded as hypothesis testing. For example, previous recommendations state that 'The results from any sub-group analyses should not be over-interpreted. Unless there is strong supporting evidence, they are best viewed as hypothesis-generating. In particular, one should be wary of evidence suggesting treatment is effective in one only sub-group' [24]. Our recommendations are aimed at researchers interested in systematic reviews and meta-analyses, although they could also be relevant for moderator analyses within trials. However, for advice on appropriate design and analysis of trials, for the testing of moderators we refer the reader elsewhere [26-28]. We note that there are several excellent published methodological papers for researchers interested in conducting moderator analysis within a trial (e.g., [28])

The recommendations from this consensus are less conservative than several other well-publicised recommendations. We compared our recommendation with the Consolidated Standards of Reporting Trials (CONSORT) statement [25]. CONSORT discusses the analysis and reporting of sub-groups, although not in reference to systematic reviews but individual trials. In this recommendation (12b), it is indicated that the analysis of sub-groups should involve a test of interaction. However, although they do not explicitly recommend against *post-hoc *sub-group comparisons, they regard these as lacking credibility. Item 18 of the CONSORT statement recommends that *a-priori* and *post-hoc* analyses are clearly indicated and differentiated in individual trial analyses. The Centre for Reviews and Dissemination (CRD) also state that planned analyses are more credible than *post-hoc* analyses. The Cochrane Handbook regards numerous *post-hoc* sub-group analyses as data dredging (item 9.6.6). The CRD recommend that few potentially important sub-group analyses are carried out to avoid false negative and false positives. In contrast, the recommendations from the current consensus were not in favour of limiting the number of sub-group analyses carried out at the meta-analytic stage, although they emphasise that the interpretation of sub-group analysis depends on a clear rationale for every sub-group reported. Authors of the The Cochrane Handbook comments that to be convincing, differences between sub-groups should be clinically plausible and supported by other external or indirect evidence (item 9.6.6). The recommendations from this consensus support this through our second assessment criteria, stating that selection of factors for analysis should be theory/evidence driven, but caution that perceived clinical plausibility is limited by the state of current knowledge.

However, there are some differences between our recommendations and those of Cochrane. The Cochrane Handbook recommends that there are adequate studies to justify sub-group analyses and meta-regressions, including at least 10 observations available for each characteristic modeled, *i.e*. 10 studies in a meta-analysis. This consensus did not support such a restriction. The Handbook also recommends that the sub-groups are specified *a-priori*based on evidence and theory. In this consensus, participants felt that applying these recommendations too strictly is not advisable because new developments may occur based on chance findings and as long as clear statements are made about whether testing of sub-groups was *a-priori *or *post-hoc*, both advance knowledge. Several panel members commented that most data is under-analysed and that this is the main reason for cautiously supporting *post-hoc *analyses. However, the consensus strongly recommends that *post-hoc *testing is regarded as exploratory and needs to be evaluated prospectively. Finally, while other guidelines advise to keep sub-group analyses to a minimum, because of the inherent problems associated with multiplicity of testing [3] we were more concerned with under-analysing data.

### The criteria

The panelists identified two criteria as necessary for reporting findings as confirmatory (see Table 3). These refer to an explicit and specific *a-priori *statement, preferably in the protocol of the included trial, describing the intended moderator analysis and the target outcome. In addition, such *a-priori *statements should be backed by theory, evidence or both, although the decision about whether such backing is sufficient is inherently subjective -- what would appear to be a convincing theory to one researcher may not be convincing to another. In ideal circumstances, such *a-priori *hypotheses would be accompanied by a sample size estimate specific for testing moderators. However, the experts who took part in this consensus study felt that this criterion was too strict and would prohibit inclusion of most trials. If the recommendations are adopted by the scientific community, and calculation of power becomes better informed by systematic reviews of previous, weaker studies, this criterion may become part of the core criteria.

**Table 3:** Criteria for inclusion in meta-analysis of moderators

Criteria	Necessary for inclusion in meta-analysis confirming moderator effects	Necessary for inclusion in meta-analysis exploring moderator effects	Criteria for the judgment of *yes*	Exceptions
Was the analysis *a- priori*	**√**		Mention of explicit hypothesis planned in protocol stating which sub-groups will be tested for which outcome	Criterion is not fulfilled in cases where the protocol includes a considerably large set of stated hypotheses or vague hypotheses (e.g. psychological factors will interact with treatment allocation')

Was selection of factors for analysis clinically plausible and either or both:	**√**		A description of theoretical background, or reference to other published evidence leading to the hypothesis	Is not fulfilled in cases where the meta-analyst considers the theory/evidence to be weak, but should not form reason for exclusion.

i) Theory based				

ii) Evidence based				

Were moderators measured prior to randomisation?	**√**	**√**	Specific statement that baseline measurement occurred prior to randomization	Not applicable for baseline factors that do not change over time, such as gender, or for cluster randomization.

Adequate quality of measurement of baseline factors	**√**	**√**	If there is published evidence to support good measurement properties of measurements for target population, according to meta-analysts' protocol.	Is not fulfilled where there is inadequate variability in baseline measure.

Contains an explicit test of the interaction between moderator and treatment	**√**	**√**	Ideally, Report a pooled effect size with 95% confidence intervals. Other acceptable analysis includes regression etc.	Not fulfilled when sub-groups are tested separately, or in excessive multiple testing.

The panelists also identified three criteria that should be applied in all cases and are necessary for inclusion of any trial in meta-analysis of moderators. The first of these states that baseline factors should be measured prior to randomisation, as they may change once allocation is known. Clearly there are some circumstances in which this criterion would not apply; for example, when baseline factors are un-modifiable (such as sex and age at randomisation). Researchers may want to consider other circumstances in which the criterion may also not apply, such as in cluster randomisation or in trials where blinding of clinicians, patients and measurers are fully realised. When cluster randomisation is used, baseline data on moderators should be collected prior to individuals becoming aware of their allocation status. The second criterion focuses on the quality of measurement of baseline factors. This is a natural extension from criteria currently applied in reference to outcome measures in systematic reviews of RCTs. Finally, the panel agreed that a specific test of the interaction between baseline factors and interventions must be presented: Ideally this would be a report of an effect size with 95% confidence interval The panel felt that insisting on this mode of presentation may be premature, but as a culture for good reporting of moderator analysis is developed this criterion may be re-visited. The presence of all five criteria allows findings to be regarded as confirmatory. The presence of the final three allows inclusion for exploratory research (see Table 3).

### Strengths and limitations

This is a consensus panel and we recognise that the search, the panel members, and the items presented are neither exhaustive nor representative of all issues surrounding moderator analysis. We recognise there is potential for bias since criteria were abstracted from published work from people who later joined the panel. However, we also invited a sub-group of people who carried out practical work in this area, but had not published methodological papers in the area, and whose work did not therefore contribute to the extracted list of items.

Our search for methodological publications in which moderator analysis was considered was limited; most common databases (PubMed and Web of Science) do not currently allow one to filter for methodological papers. This limitation should be addressed by responses to the recommendations, and we hope to initiate an iterative process leading to amendments and further developments of recommendations.

Some of the criteria extracted from methodological publications described processes that are not usually carried out in the context of RCTs. For example, criteria *4b*, (did the authors explore residual variances of interactions when carrying out multiple two-way interactions?). Our panelists were uncertain about the necessity of applying these criteria, although one panelist commented that such exploration would be informative in understanding power issues. We therefore particularly welcome comments about items about which the panel felt uncertain.

Several of the criteria are inherently subjective in that they are derived from opinion and judgments of the meta-analyst. This is true for many methodological criteria and we urge meta-analysts to give explicit details of the protocol they employed in making their decisions. We note also, that ascertaining whether the rationale for sub-group analysis through a plausible theory may be particularly difficult in areas where theories are abundant.

We recognise that the criteria proposed are only the first step in helping researchers decide on studies for inclusion in meta-analysis of interactions. We note that even if the criteria from this consensus are applied carefully, the analysis could still be compromised by the RCTs providing insufficient data, using different statistical models, having different covariates in the model, and measuring the moderator and outcome with different instruments. Thus, there is still a need to develop methodological criteria to guide meta-analysis of moderators, including statistical guidance for the analysis of interactions.

## Conclusions

Pooling information from trials that have carried out sub-group analysis based on similar baseline factors is reasonable, and may be useful even when based on few trials. Exploration of treatment effect moderators could point to certain patients being better suited to certain interventions. This consensus proposes a set of criteria to assist researchers engaged in such analysis.

There is consensus from a group of 21 international experts that methodological criteria to assess moderators within systematic reviews of RCTs are both timely and necessary. The consensus from the experts resulted in five criteria divided into two groups when synthesising evidence: confirmatory findings to support hypotheses about moderators and exploratory findings to inform future research. The criteria could be tested empirically, for example, by comparing results of moderator subgroup analysis based on published effect sizes following the criteria to results of moderator subgroup analysis obtained through individual patient data meta-analysis of the same papers. In addition, the findings from reviews using these criteria could be compared to the findings from reviews that followed the guidance from the Cochrane handbook and the COSORT recommendations.

## Competing interests

The authors declare that they have no competing interests.

## Authors' contributions

TP directed the project, was involved in data extraction at all stages and wrote the first draft of the manuscript. CM was responsible for search co-ordination with the consensus panel and writing of the manuscript. RF was responsible for carrying out the data analysis using the RAND/UCLA appropriateness method and commented in detail on successive drafts of the manuscript. MU, DC and ST were members of the research team, overseeing the project and contributed to the quality assessment of data extraction and commented on successive drafts of the manuscript. All authors read and approved the final manuscript.

## Appendix: Details of RAND methodology

For each item, the median score and the inter-quartile range were calculated. The 30th and 70th percentiles were calculated to give the inter-percentile range (IPR) and the inter-percentile range adjusted for symmetry (IPRAS) was also calculated. The IPRAS is the threshold beyond which the IPR for a particular item indicates disagreement. Using the IRPAS and the IPR to judge disagreement reproduces 'classic' RAND definitions of disagreement when used on panels made up of multiples of three, but has the advantage that it may be applied to panels of any size [20].

Calculations are based on the following formula:

$$IPRAS=IPR_{r}+(AI\times CFA)$$

*Where IPR*_*r *_*is the inter-percentile range required for disagreement when perfect symmetry exists, AI is the asymmetry index, and CFA is the correction factor for asymmetry*.

In this formula, optimal values for IPR_r _and CFA of 2.35 and 1.5 have been derived following empirical work on a 9-point scale. Fitch et al explain that these best approximate 'classic' RAND definitions of agreement [20]. The IPRAS threshold is dependent on the symmetry of ratings about the median. Thus, each item requires a different IPRAS to be calculated. Consequently, indication *i *is rated with disagreement if the IPR_*i *_> IPRAS_*i*_. We calculated the ratio of these: The disagreement index [21]. If the disagreement index is less than 1.0, we considered that there was no disagreement for the item in question. An item was considered 'appropriate' for inclusion if its median rating, M, was ≥6, 'uncertain' if 3 ≤ M < 6, and 'inappropriate' if M < 3.

## Pre-publication history

The pre-publication history for this paper can be accessed here:

http://www.biomedcentral.com/1471-2288/11/14/prepub
